# IGF1R signalling is a guardian of self-tolerance restricting autoantibody production

**DOI:** 10.3389/fimmu.2022.958206

**Published:** 2022-08-29

**Authors:** Malin C. Erlandsson, Seval Erdogan, Caroline Wasén, Karin M. E. Andersson, Sofia T. Silfverswärd, Rille Pullerits, Mats Bemark, Maria I. Bokarewa

**Affiliations:** ^1^ Department of Rheumatology and Inflammation Research, Institute of Medicine, University of Gothenburg, Gothenburg, Sweden; ^2^ Rheumatology Clinic, Sahlgrenska University Hospital, Gothenburg, Sweden; ^3^ Ann Romney Center for Neurologic Diseases, Harvard Medical School, Brigham and Women’s Hospital, Boston, MA, United States; ^4^ Department of Biology and Biological Engineering, Chalmers University of Technology, Gothenburg, Sweden; ^5^ Department of Clinical Immunology and Transfusion Medicine, Region Västra Götaland, Sahlgrenska University Hospital, Gothenburg, Sweden; ^6^ Department of Microbiology and Immunology, Institute of Biomedicine, Sahlgrenska Academy, University of Gothenburg, Gothenburg, Sweden

**Keywords:** rheumatoid arthritis, FoxO1, IGF1R, marginal zone, autoantibodies

## Abstract

**Objective:**

Insulin-like growth factor 1 receptor (IGF1R) acts at the crossroad between immunity and cancer, being an attractive therapeutic target in these areas. IGF1R is broadly expressed by antigen-presenting cells (APC). Using mice immunised with the methylated albumin from bovine serum (BSA-immunised mice) and human CD14^+^ APCs, we investigated the role that IGF1R plays during adaptive immune responses.

**Methods:**

The mBSA-immunised mice were treated with synthetic inhibitor NT157 or short hairpin RNA to inhibit IGF1R signalling, and spleens were analysed by immunohistology and flow cytometry. The levels of autoantibody and cytokine production were measured by microarray or conventional ELISA. The transcriptional profile of CD14^+^ cells from blood of 55 patients with rheumatoid arthritis (RA) was analysed with RNA-sequencing.

**Results:**

Inhibition of IGF1R resulted in perifollicular infiltration of functionally compromised S^256^-phosphorylated FoxO1^+^ APCs, and an increased frequency of IgM^+^CD21^+^ B cells, which enlarged the marginal zone (MZ). Enlargement of MHCII^+^CD11b^+^ APCs ensured favourable conditions for their communication with IgM^+^ B cells in the MZ. The reduced expression of ICOSL and CXCR5 by APCs after IGF1R inhibition led to impaired T cell control, which resulted in autoreactivity of extra-follicular B cells and autoantibody production. In the clinical setting, the low expression of IGF1R on CD14^+^ APCs was associated with an involuted FOXO pathway, non-inflammatory cell metabolism and a high IL10 production characteristic for tolerogenic macrophages. Furthermore, autoantibody positivity was associated with low IGF1R signalling in CD14^+^ APCs.

**Conclusions:**

In experimental model and in patient material, this study demonstrates that IGF1R plays an important role in preventing autoimmunity. The study raises awareness of that immune tolerance may be broken during therapeutic IGF1R targeting.

## Introduction

Insulin-like growth factor 1 receptor (IGF-1R) is a trans-membrane tyrosine kinase expressed in almost every mammalian cell. This is one of the most potent signalling axis that control cell survival and apoptosis. Ligation of IGF1R, primarily by IGF-1 or insulin, leads to the phosphorylation of Insulin receptor substrates 1 and 2 (IRS1 and IRS2), two intrinsic tyrosine kinases that recruit downstream effectors for the PI3-kinase and mitogen activated protein (MAP) kinase signalling cascades. Phosphorylation of IRS enables docking of SH2 containing proteins including PI3K, SHP2, ERK and Grb2 (1), subsequently leading to the activation of the of serine/threonine protein kinase Akt (protein kinase B) and the MAP kinases. Inhibitory signals from IGF1R are mediated by inhibition of the FOXO transcription factor family and are important for coping with oxidative stress, senescence, and autophagy (2). Studies in tissue-specific experimental models revealed a central role for IGF1R signalling in regulating glucose metabolic functions in the liver, skeletal muscles, and adipose tissue (3). In other tissues, IGF1R is important for homeostasis, reparatory ability, and resistance to stress. IGF1R signalling dysfunction in the brain results in neurodegeneration and contributes to the pathology of the Alzheimer’s, Parkinson’s, and Huntington’s diseases (4). In the lung tissue, deletion of IGF1R altered myofibroblast differentiation and mechanosensory ability (5).

In addition to healthy tissues, IGF1R is commonly over-expressed, and the signalling pathway constitutively activated in numerous cancers contributing to mesenchymal transition of stroma cells and malignant development (6, 7). In cancer cells, IGF1R participates in the control of cellular processes ranging from proliferation and migration to drug resistance (8, 9). Many of these effects are attributed to the function of IGF1R in cell-matrix and cell-cell interactions through its binding to the adhesion signalling complex (10), which promotes a strengthened signal from the focal adhesion kinases and casein kinase. In synergy with nuclear receptors to oestrogen, epidermal growth factor and thyrotropin, IGF1R contributes to the long-term and ligand-independent activation of those receptors in the nucleus (3).

IGF1R acts at the crossroad between cancer and immunity. IGF1Ris broadly expressed in macrophages and, under physiological conditions, sufficient IGF1R signalling keeps the balance between pro- and anti-inflammatory activities of these cells (11). Studies have demonstrated that conditional deletion of IGF1R in myeloid progenitors results in the upregulation of the key anti-inflammatory markers in macrophages. Upon pathogen challenge, IGF1R-deficient macrophages exhibited reduced accumulation in lesions and poor phagocytic ability, which delayed resolution from infection (11, 12). Inflammation induces significant changes in IGF1R signalling by limiting the production and availability of IGF1 ligand (13, 14) and by enabling inhibition of the IRS adaptor molecules, which slows down the signal propagation (15). This causes a high reciprocal expression of IGF1R and triggers proliferation and survival of macrophages at the site of inflammation. IGF1R signalling in leukocytes is associated with activation of STAT3 and STAT5 proteins, which initiates dominant expression of RORγt and NF-κB transcription factors and acquisition of the effector phenotype of Th17 cells (16, 17). Such a shift in the regulatory balance of T cells following IGF1R inhibition has been recognised in several autoimmune conditions including multiple sclerosis (18, 19), rheumatoid arthritis (20, 21) and type I diabetes mellitus (22, 23). Together, this points out the important role of IGF1R plays in the pathogenesis of aberrant T cell self-recognition. A different mode of IGF1R dependent autoimmunity has been described in thyroiditis, where IGF1R bearing B cells are responsible for the production of pathogenic antibodies against the thyrotropin receptor (24).

Checkpoint inhibition of immune cells often potentiates anti-tumour immunity. Thus, it is not surprising that therapeutic targeting of the IGF1R axis is under continuous investigation both for solid tumours and haematologic malignancies (25). In non-malignant condition, the therapeutic potential of IGF1R inhibition was assessed during autoimmune inflammation (16, 17, 26, 27). The arsenal of IGF1R intervention includes monoclonal antibodies, small inhibitory molecules, microRNA, and antisense oligomeric peptides targeting intracellular downstream mediator proteins (28). Treatment with check-point inhibitors in cancer is associated with a high frequency of autoimmune adverse events, which is not surprising given that their primarily targets are T cell co-receptors (29, 30). Cancer patients with pre-existing autoimmune diseases are usually excluded from clinical trials with the checkpoint inhibitors due to this increased risk of toxicity. This patient group cannot fully benefit from advances in biologic anti-cancer therapy. This may be particularly relevant for IGF1R that is expressed in a much broader variety of cells, with off-target effects are likely to occur in many cell types including B cells.

In this study, we investigated a connection between IGF1R signalling and self-tolerance. We used a synthetic small molecule and a short hairpin RNA to inhibit IGF1R signal in mBSA-immunised mice and studied changes in the marginal zone (MZ) and localisation of IRS1 and FOXO1 molecules downstream of IGF1R. We also investigated changes in populations of antigen-presenting cells (APCs) and B cells, and the cytokine and antibody production by splenocytes in response to IGF1R inhibition. In RA patients, we used RNA sequencing to explore an association between autoantibody positivity and IGF1R expression and signalling in antigen presenting CD14^+^ cells.

## Materials and methods

### Patient material

Blood samples were collected from 55 randomly selected female patients with RA disease visiting the Rheumatology Clinic at Sahlgrenska University Hospital, Gothenburg, Sweden, during the period October 7^th^, 2019, and October 26^th^, 2020. All RA patients fulfilled the 2010 American College of Rheumatology classification criteria for RA (31). Clinical characteristics of the patients are presented in **Table 1**. At time of blood sampling, the patients were on average 60.9 years old, and had a mean disease duration of 13 years. Thirty-four patients (61.8%) had therapy with methotrexate (MTX), 20 patients with JAK-inhibitors, 20 patients with biologics, and 29 patients (52.7%) with a combination of several disease-modifying anti-rheumatic drugs (DMARDs). At the time of blood sampling, all patients underwent a clinical assessment of their joints and they completed the Stanford Health Assessment Questionnaire disability index (HAQ) (32). Disease activity score was calculated based on examination of the 28 tender and swollen joints (DAS28) and erythrocyte sedimentation rate.

**Table 1:** Clinical characteristics of the patients with rheumatoid arthritis used as a source of CD14^+^ cells.

	IGF1R high*, n=28		IGF1R low, n=27
Age, y	56.2 ± 11.8	p=8.8e-4	65.8 ± 7.4
Disease duration, y	12.4 ± 8.8	–	14.3 ± 11.3
DAS28	2.60 ± 1.09	–	2.74 ± 1.07
IGF1, microgram/L, serum	123 ± 51	–	114 ± 30
**Autoantibodies (n)**			
None	12	p=0.0099	3
RF	13	p=0.0084	22
Anti-CCP	14	–	16
**Treatment (n)**			
MTX	12	p=0.0039	22
Biologics	7	–	13
JAK-inhibitors	19	p=3.8e-7	1
Oral corticosteroids	6	–	3

Mean ± standard deviation

*Split by median IGF1R in CD14+ cells

The study was approved by the Swedish Ethical Review Authority (Dnr. 2019-03787) and conducted in accordance with national regulations and the International Conference on Harmonization Good Clinical Practice requirements, based on the Declaration of Helsinki. All patients provided written informed consent before subjected to any study-related procedures.

### Cell isolation and culturing

Blood samples from RA patients were obtained from the cubital vein directly into heparinized vacuum containers for further isolation of cells. Human peripheral blood mononuclear cells were isolated using density gradient separation on Lymphoprep (Axis-Shield PoC As, Norway). CD14^+^ cells were isolated using positive selection (Stemcell Technologies 17858). Control of the isolated cells by flow cytometry demonstrated 78-90% purity of CD14^+^ cells. The cells were seeded (1.25x10^6^ cells/ml) and stimulated for 2 h with lipopolysaccharide (LPS) 5µg/ml (Sigma-Aldrich, Saint Luis, Missouri, USA) in RPMI medium supplemented with 50µM β_2_-mercaptoethanol (Gibco, Waltham, Massachusetts, USA), Glutamax 2mM (Gibco), gentamicin 50µg/ml (Sanofi-Aventis, Paris, France) and 10% foetal bovine serum (Sigma-Aldrich) at 37°C in a humidified 5% CO_2_ atmosphere. Supernatants were collected for measurements of soluble proteins and cells were collected for RNA preparation.

### RNA preparation and whole-genome transcriptomic analysis

RNA from CD14^+^ cell cultures was prepared using the Total micro mRNA kit (Norgen, Ontario, Canada). Quality control was performed by Bioanalyzer RNA6000 Pico on Agilent 2100 (Agilent, St.Clara, CA, USA). Deep sequencing was done by RNAseq (Hiseq2000, Illumina) at the BEA Core Facility, Karolinska Institute, Sweden. Raw sequence data were obtained as Bcl-files and converted into fastq text format using the bcl2fastq program from Illumina. Validation of RNAseq was performed using qRT-PCR as described below. Fastq-files and raw reads are deposited in Gene Expression Omnibus at the National Center Biotechnology Information with the accession code *GSE201670*.

### RNA-sequencing analysis

Mapping of transcripts to the genome was done using the Genome UCSC annotation set for hg38 human genome assembly. As the samples had been sequenced in two batches, we performed a batch correction with ComBat_seq from the sva package [R package version 3.44.0 (33)]. The counts data were filtered for the genes that had more than 5 counts in at least one third of the samples and scaled by size factors with DESeq2. Differentially expressed genes (DEGs) were identified by R-studio using Benjamini-Hochberg adjustment for multiple testing (Bioconductor package, “DESeq2” version 1.26.0). Volcano plots were constructed with “EnhancedVolcano” (version 1.4.0).

### IGF1R signalling score

The IGF1 signalling score was built based on the publicly available transcriptomic RNA-seq data set of human monocyte-derived macrophages stimulated *in vitro* with IGF1 [*GSE202515* (34)]. We extracted the data of macrophages stimulated with IGF1 and used the cultures stimulated with BSA-PBS as control. The counts data was filtered for genes that had more than 5 counts in at least one third of the samples and scaled by size factors with DESeq2 (35). Differential expression was calculated with DESeq2 and all genes with an adjusted p-value (BH) below 0.05 were used to construct an IGF1 signalling gene set.

We next extracted the IGF1 signalling gene set from the *GSE201370* data set, log-transformed the normalised counts and calculated z-scores for each gene. All genes that were predicted to be downregulated were multiplied with -1 (so that a positive score would always indicate more signalling). Lastly, the IGF1-score was defined as the average z-score in each sample of the *GSE201370* data set. All statistics were performed in R version 4.1.2 and Prism 9 for macOS version 9.3.1.

### Animal model

**Figure 1:**
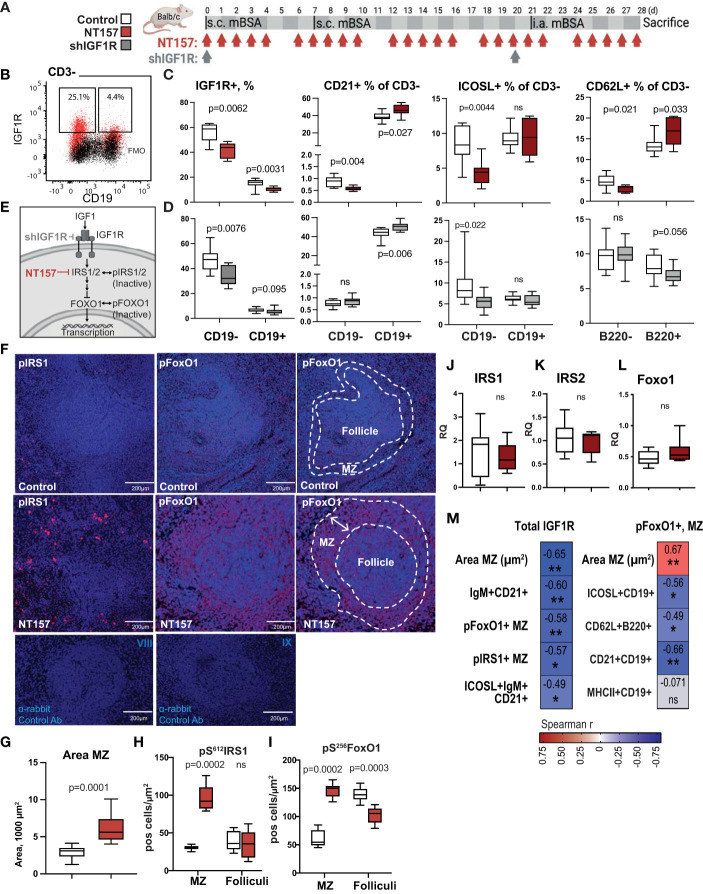
Enlargement of MZ following inhibition of IGF1R signalling. **(A)** IGF1R-targeting experimental setting using NT157 and shIGF1R treatment. **(B)** Flow cytometry gating strategy for IGF1R^+^ subsets of CD19^-^ and CD19^+^ leukocytes in spleen. **(C)** Box plots of IGF1R^+^ subsets in NT157-treated mice and **(D)** in shIGF1R-treated mice. **(E)** IGF1R signalling. **(F)** Confocal images of pS^612^IRS1^+^ and pS^256^FOXO1^+^ cells in MZ of control and NT157-treated mice. **(G)** Box plots of MZ area. **(H)** Box plots of pIRS1^+^ and **(I)** pFOXO1^+^ cell density in MZ and follicles. **(J–L)** Box plots of mRNA IRS1, IRS2 and FOXO1 levels in spleen of NT157-treated mice. **(M)** Correlation matrix for total IGF1R^+^ cell frequency and area MZ, pFoxo1^+^, pIRS1^+^ cells in MZ. Spearman correlation was applied. *p<0.05, **p<0.01; ns, not significant..

In total, 38 female Balb/c mice (Charles River, Scanbur, Karlslunde, Denmark) were at 8 weeks of age subjected to antigen challenge using methylated albumin from bovine serum (mBSA). On day 0 of the experiment, the mice were immunised subcutaneously with mBSA, on day 7 they received a booster with mBSA subcutaneously, and on day 21 mBSA was injected intra-articularly in the knee joint. On day 28, the mice were sacrificed (**Figure 1A**). All mice were housed at the Department of Rheumatology and Inflammation Research (Gothenburg University) in groups of 5-10 mice per cage and under standard conditions at 20-26°C and with a 10-14 hours light-dark cycle. They were fed standard chow (7% simple sugars, 3% fat, 50% polysaccharide, 15% protein, energy 3.5kcal/g, VWR International) and water *ad libitum*. The Gothenburg University guidelines for the care and use of laboratory animals were strictly followed. The experiments were approved by the Animal Ethical Board at the University of Gothenburg.

### Intervention

Two different approaches were used to inhibit IGFR1 signalling in mice. In the first approach, IGF1R signalling was disrupted *via* inhibiting IRS1/2 by subcutaneous injections with the synthetic molecule NT157 (NovoTry Therapeutics Ltd., Israel) at doses 10 mg/kg dissolved in 2-Hydroxypropyl-β-cyclodextrine (20% in ddH_2_0, Sigma-Aldrich). The control group of mBSA-immunised mice was treated with the identical volume of 2-Hydroxypropyl-β-cyclodextrine (20% in ddH2O). In the second approach, IGF1R was inhibited using lentiviral particles (MISSION TRCN000023490 and TRCN0000023493, Sigma-Aldrich) containing shRNA that target IGF1R (shIGF1R). Control mice received transduction particles (SHC002H) producing non-targeting RNA (shNT). Throughout the course of each experiment, mice were individually monitored every day by assessment of general condition and total body weight. At the end of the experiment, the mice were sacrificed, and spleen and blood samples were collected. Spleens were fixed in 4% formaldehyde for 48 h, and serum was collected from blood after centrifugation.

### Histology and immunohistochemistry

Paraffin sections (4 μm) of spleens were deparaffinised, subjected to epitope retrieval with Diva Decloaker (Biocare Medical, Concord, CA, USA), and non-specific binding was blocked with goat serum (5%, Thermo Fisher Scientific). After incubation with pS^612^IRS1 antibody (1:500; 44-550G, Invitrogen), pS^256^FOXO1 antibody (1:100; PA5-38132, Invitrogen), biotin-SP-conjugated F(ab’)2 goat anti mouse IgM antibody (1:100; 115-067-020 Jackson Immunoresearch), or rabbit gamma globulins (Jackson ImmunoResearch Laboratories, West Grove, PA, USA) as a negative control at +4°C overnight, the specimens were incubated with Alexa flour-conjugated 488 goat anti-rabbit IgG (1:250; Thermo Fisher Scientific) for 1 h at room temperature, or Streptavidin-Alexa Fluor 635 (1:250; Thermo Fisher Scientific) for 20 min. pS^612^IRS1 sections were subjected to biotinylated peanut agglutinin (PNA, 1:250; Vector) staining for 30 min at room temperature followed by Streptavidin-Alexa Fluor 635 (1:250; Thermo Fisher Scientific). In all sections autofluorescence was blocked with 0.5% Sudan Black B (Sigma) dissolved in 70% ethanol, and nuclei were stained with Hoechst 34580 (1:100; Thermo Fisher Scientific) and haematoxylin. Finally, the sections were mounted with Prolong Gold Antifade Mountant (Prolong Gold Antifade Mountant).

### Confocal imaging

Images of spleen sections were captured with a LSM 700 confocal microscope (Carl Zeiss), using 10× or 20× objectives and ZEN 2012 software (Carl Zeiss). The morphometric analysis was blinded and performed with CellProfiler software (version 3.1.9, Broad Institute). Objects were measured in 12-bit composite images with threshold adjusted individually for each staining series, to optimise the software’s ability to identify individual positive cells. pS^612^IRS1^+^, and pS^256^FoxO1^+^ cells were counted automatically using the IdentifyPrimaryObjects pipeline. The follicles and marginal zone area were identified using the IdentifyPrimaryObjects and IdentifyObjectsManually pipelines, respectively. The measurements were given in pixels and converted into µm^2^ using the pixel-to-micrometre conversion factor.

### Flow cytometry

Spleen tissue was grinded through a 70-µm filter and red blood cells were lysed with a 0.83% NH4Cl lysis buffer. The cells were then washed and suspended in FACS buffer (PBS, 10% FBS, 0.09% NaN3, 0.5 mM EDTA). Next, the cells were pre-incubated with Fc-block (BD Bioscience). Cells were stained with specific antibodies (listed in *SI Appendix*, **Table S1**). IGF1R was visualised with Alexa647-conjugated anti-rabbit IgG (#4414, Cell Signalling) and intracellular staining kit (eBiosciences). Flow cytometry was performed with a Becton Dickinson (BD) FACS Canto II. Analysis was done with FlowJo and gating of the cells was based on the isotype control or on the fluorochrome minus one (FMO) setting.

### Antibody measurement

Rheumatoid factor (RF) and anti-citrullinated protein antibodies (ACPA) were measured in serum samples at the accredited laboratory of Clinical Immunology at Sahlgrenska University Hospital. ACPA was measured using an automated multiplex method (anti-CCP2, BioRad BioPlex 2200, Hercules, CA). Positive cut-off points were set according to the manufacturer at above 3.0 U/ml. Antibodies against Fc-region of gamma globulin (total RF) were measured by rate nephelometric technology (Beckman Immage 800, Beckman Coulter AB, Brea, CA) with a cut-off for RF positivity at 20 U/ml.

In mouse samples, antigen specific antibodies to mBSA, and antibodies to Fc-region of IgG (RF) were measured using ELISA as described previously (36). Essentially, plates were coated with mBSA (10 µg/mL) or with goat F(ab´)_2_ fragments to mouse Igs (5 μg/mL). Anti-CCP antibodies were measured using antigen-coated plates from Immunoscan CCPlus (Euro-Diagnostica, Malmö, Sweden). Antibodies to double stranded (ds)DNA were measured using the plates coated with 20 μg/ml poly-L-lysine, then with 20 μg/ml boiled and snap-chilled calf thymic DNA as described (37). Binding of Igs to antigen was detected using biotin-conjugated antibodies specific for IgM, IgG, IgG1, IgG2a, IgG2b, and IgG3 followed by extravidin–HRP (Sigma) and 3,3′, 5,5′ Tetramethylbenzidine substrate. Measurements were performed using a serial dilution of serum and supernatant; the absorption was read at 450 nm. Antibodies and reagents used for ELISA are listed in *SI Appendix*, **Table S2**.

### Gene expression analysis by conventional RT-PCR

The mRNA from murine spleen cells was extracted using RNeasy Mini Kit (Qiagen, Valencia, CA). The complementary DNA was prepared using High-Capacity cDNA Reverse Transcription Kit (Applied Biosystems, Foster city, CA). Quantitative PCR was performed with SYBR Green qPCR Mastermix (Qiagen) using a ViiA™ 7 Real-Time PCR System (Applied Biosystems). The level of mRNA for *Foxo1*, *Irs1*, *Irs2* and *Marco* genes was measured. Expression levels of these genes were normalised to the reference gene, *Gapdh* (TATAA Biocenter, Sweden). The primer pairs can be found in *SI Appendix*, **Table S3**. Melting curves for each PCR were performed between 60°C and 95°C to ensure specificity of the amplified product. The results were expressed as the fold change compared with the expression levels in the control cells using the ddCt-method.

### Statistical analysis

Statistical significance of the differences was determined using non-parametric Mann-Whitney U-test as they were previously tested for normality and the data was not normally distributed. Spearman’s correlation coefficient was used for correlation analysis. Graphing and statistical analyses were conducted using GraphPad Prism (version 9.0 for Mac; GraphPad Software). P-values <0.05 were considered to represent statistical significance.

### Data availability

Fastq-files and raw reads are deposited in Gene Expression Omnibus at the National Center Biotechnology Information (NCBI) with the accession code *GSE201670*. Other data that support the findings of this study are available upon reasonable request and by contacting the corresponding author.

## Results

### Inhibition of IGF1R results in the enlargement of the MZ area

To assess the role of IGF1R signalling in leukocytes, we treated the mBSA-immunised Balb/c mice with IGF1R inhibitors NT157 or shIGF1R (**Figure 1A**). The synthetic molecule NT157 induces a conformational change of the IGF1R that weakens IRS binding (38) thereby inhibiting IGF1R signalling, while shIGF1R directly diminishes translation of IGF1R. We then analysed IGF1R expression levels on CD3^-^CD19^-^ antigen presenting cells (APC) and on CD3^-^CD19^+^B cells in the spleen by flow cytometry (**Figures 1B**). This analysis demonstrated that the inhibition of IGF1R in these populations was achieved by both NT157 treatment (**Figures 1C**) and by shIGF1R (**Figure 1D**).

We investigated the spleen morphology of NT157-treated and sham-treated control mice with the haematoxylin and eosin staining and found that the MZ area (µm^2^) around the follicles was significantly enlarged after treatment with NT157 as opposed to the sham controls (p=9.1e-5, **Figures 1F, G**). Since the inhibition of IGF1R led to a change in the MZ area, we assessed the impact of the IGF1R signalling on CD21^+^ cells particularly present in the MZ, by flow cytometry. Here, CD21 expression was found on less than 1% of APC and showed a diverse response in NT157 and shIGF1R treated mice (**Figures 1C, D**). On the contrary, CD21 expression were abundant in CD19^+^ B cells and CD21^+^CD19^+^ B cells were further significantly enriched after the inhibition of IGF1R signalling by NT157 and by shIGF1R treatment (**Figures 1C, D**).

IGF1R signalling is critically dependent on the activity of the adaptive molecules IRS1/IRS2 and the FOXO family of transcription factors (**Figure 1E**). Next, a deep histological analysis of intracellular IGF1R signalling in spleen of arthritis mice was performed by staining IRS1 and FOXO1. A morphometric analysis of spleen tissue showed that IRS1, in its inactive form *i.e.* phosphorylated at serine 612 (pS^612^IRS1), was enriched within the area of MZ after IGF1R inhibition (p=0.0002, **Figures 1F–H**). In the reverse, we found no significant difference in the number of pS^612^IRS1^+^ cells in the spleen follicles after IGF1R inhibition (p= 0.72).

FoxO1 is necessary for the regulation of APC response to stimuli (39) and balance of mature B-cell subsets (40). Transcriptionally inactive FoxO1, phosphorylated at serine 256 (pS^256^FoxO1), was found abundantly within the MZ after IGF1R inhibition compared to the sham controls (p=0.0002, **Figures 1F, G**). Additionally, we found that NT157-treated mice displayed a change in the localisation pattern of pS^256^FoxO1^+^ cells. In NT157-treated mice, the pS^256^FoxO1^+^ cells were present outside the follicle and formed a ring-like structure around the follicle that corresponded to the MZ area (**Figures 1F–I**). In the sham-treated controls, pS^256^FoxO1^+^ cells were present within the follicles, dispersed within the whole follicle area. In fact, we found a significant negative correlation between the frequency of IGF1R^+^ single cells detected by flow cytometry and IHC findings in spleen, including the area of the MZ (μm^2^), the density of pS^256^pFoxO1^+^ cells, the density of pS^612^IRS1^+^ cells within the MZ, and the frequency of the IgM^+^CD21^+^B cells (**Figure 1M**). In spleen sections, we could not find colocalisation of pS^256^FOXO1 and pS^612^IRS staining suggesting that pS^256^FOXO1 and pS^612^IRS were not expressed by the same cell. This assumption is supported by the colocalisation observed between IgM and pS^612^IRS1 and lack of colocalisation between IgM and pS^256^FOXO1. The quantitative mRNA levels of FoxO1, IRS1 and IRS2 mediators downstream of IGF1R demonstrated that inhibition of IGF1R had no significant effect on their expression in spleen tissue (**Figures 1J–L**).

### Inactivation of FOXO1 in MZ reduced expression of ICOSL on MHCII+ cells

**Figure 2:**
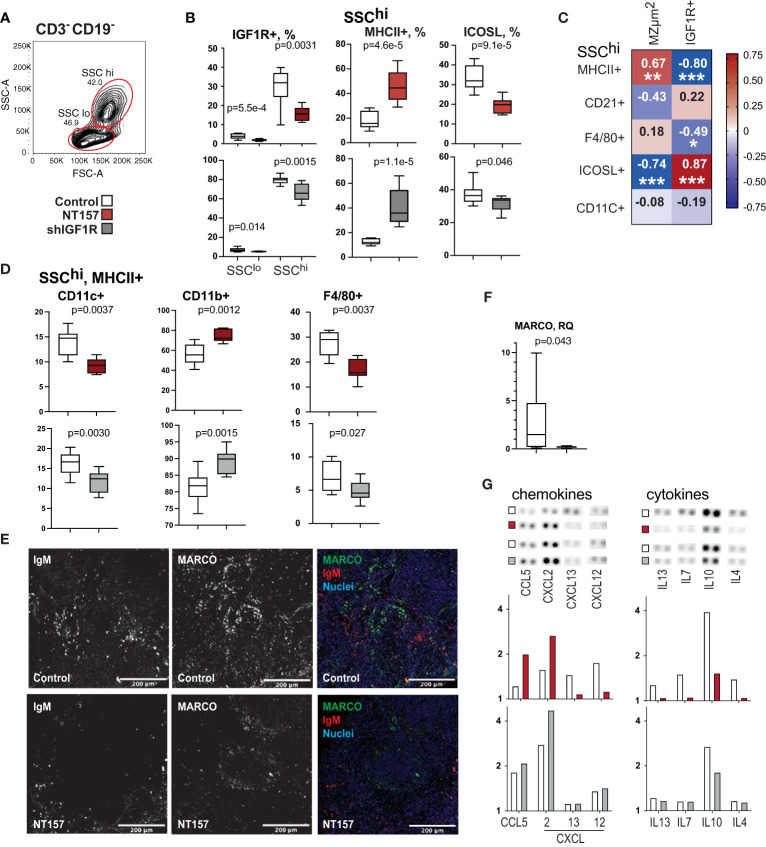
Phenotype and function of antigen presenting cells after inhibition of IGF1R signalling. **(A)** Flow cytometry gating strategy for large granular CD3-CD19- (SSC^hi^) cells by forward scatter (FSC) and side scatter (SSC). **(B)** Box plots of IGF1R+ subset in SSC^hi^ cells in NT157 and shIGF1R-treated mice. SSC^hi^ cells in NT157 (upper row) and shIGF1R (lower row) treated mice by MHCII, ICOSL, CD11b and CD11c. **(C)** Correlation heatmap between SSChi populations and MZ area and IGF1R+ cells. Spearman correlation was applied. **(D)** Box plots of CD11c^+,^ CD11b^+^ and F4/80^+^ subsets of SSC^hi^MHCII^+^ cells. **(E)** Confocal image of IgM and MACRO staining in spleen of control and NT157 treated mice. **(F)** Box plot of mRNA levels of MACRO in spleen of NT157-treated and control mice. **(G)** Dot blot array of chemokine and cytokine levels in supernatants of LPS-stimulated spleen cell cultures of NT157-treated and control, and shIGF1R-treated and control mice. *p<0.05, **p<0.01, ***p<0.001.

To further investigate APC, which are characterised by their larger cell volume and higher granularity than other cells, these cells were identified among CD3^-^CD19^-^ splenocytes by using the flow cytometry side and forward scatter parameters (SSC^hi^ cells) (**Figure 2A**). The SSC^hi^ cells expressed high levels of IGF1R and were sensitive to the IGF1R reducing effects of NT157 and shIGF1R treatments (**Figure 2B**). In SSC^hi^ population in sham-treated mice, 12-20% of the cells were MHCII^+^APC. This population expand and made up a 2-fold larger proportion after IGF1R inhibition using either NT157 or shRNA treatment. Notably, the relative frequency of the MHCII^+^SSC^hi^ population correlated strongly to the area of the MZ (r=0.67, p=0.0023) and inversely to the total IGF1R expression (r=-0.80, p=8e-4) (**Figure 2C**). MHCII^+^SSC^hi^ APC consisted of 80% of CD11b^+^ cells, and their frequency further increased after shIGF1R treatment (**Figure 2D**). In contrast, the frequency of CD11c^+^ and F4/80^+^ MHCII^+^SSC^hi^ APCs showed a decrease.

Since IGF1R inhibition resulted in MZ infiltration of APC expressing FOXO1 that lost their ability to upregulate transcription, we studied changes in the inducible costimulatory ligand (ICOSL) and L-selectin (CD62L) that are transcriptional targets of FOXO1 (41, 42). Flow cytometric analysis revealed that ICOSL expression decreased by 55% on the SSC^hi^ APC after IGF1R inhibition (**Figure 2B**), whereas IgM^+^CD19^+^ B cells reduced less than 1% (**Figure 1C**). Interestingly, the reduced frequency of ICOSL^+^ cells was strongly correlated with decreased expression in IGF1R (r=0.70, p=0.0071) (**Figure 2C**). This argues for the notion that FOXO inactivation was primarily achieved in APC.

To study the association between localisation of IgM^+^ B cells in the MZ and changes in the IGF1R signalling molecules pS^256^pFoxO1 and pS^612^IRS1, we performed co-staining of spleen section for these markers. IHC staining demonstrated the peri-follicular accumulation of IgM^+^ cells in NT157-treated mice compared to sham-treated controls (**Figure 2E**). The IgM staining in the peri-follicular areas was more likely to co-localise with pS^612^IRS1^+^ cells rather than with pS^256^pFoxO1. The macrophage population expressing the macrophage scavenger receptor with collagenous structure (MARCO) defines the MZ boundary (43). Staining for MARCO in spleen sections revealed the preserved formation of MARCO^+^ structures around follicles in sham-treated mice, while MARCO staining was less defined in NT157-treated mice (**Figure 2E**), which could correspond to a significantly reduced level of MARCO mRNA after IGF1R inhibition (**Figure 2F**). Notably, MARCO^+^ structures were found in the proximity of IgM^+^ cells in sham-treated mice and in the areas free from IgM^+^ cells in NT157-treated mice.

Since ICOSL-ICOS interaction stimulates the secretion of many cytokines (44), we analysed supernatants of LPS-activated splenocyte cultures for cytokine and chemokine production using microarrays (**Figure 2G**). Inhibition of IGF1R by NT157 and shRNA resulted in a decreased production of IL10, and IL4 cytokines that mediated communication with lymphocytes and in an increased production of chemokines CCL5/RANTES, and CXCL2 that are required for B cell recruitment to the MZ (45).

### Deficient IGF1R signal in the MZ results in accumulation of ICOSL-IgM+ B cells

**Figure 3:**
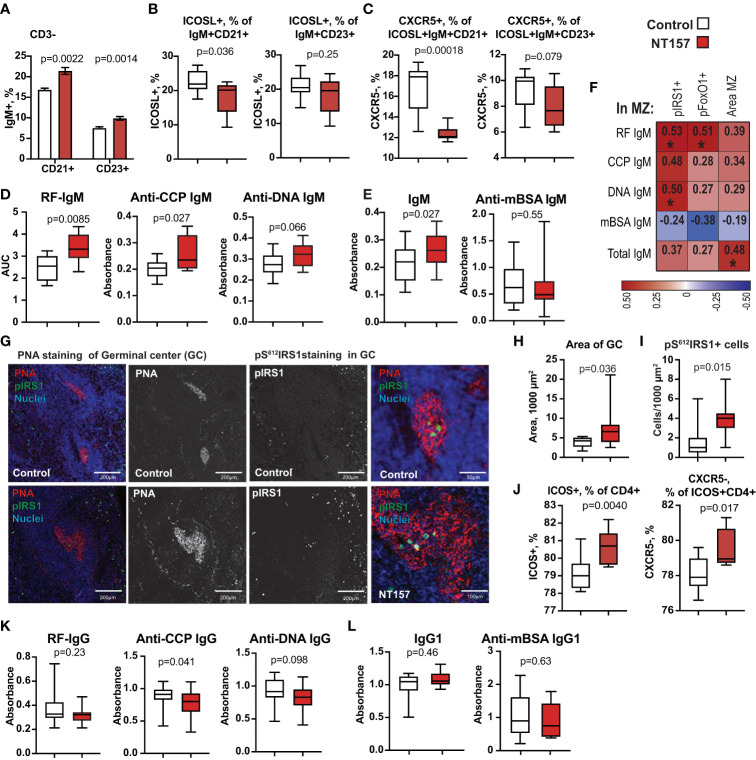
Changes in IgM^+^CD19^+^ B cells and function after inhibition of IGF1R signalling. **(A)** Bar plots of changes in CD21 and CD23 expression on IgM^+^CD19^+^ cells. **(B)** Box plots of frequency of ICOSL on CD21^+^ and CD23^+^ B cells. **(C)** Box plots of frequency of CXCR5 on ICOSL^+^CD21^+^ and ICOSL^+^CD23^+^ IgM^+^CD19^+^ cells. **(D)** Box plots of IgM antibodies against RF, CCP, dsDNA. **(E)** Box plots of total IgM and anti-mBSA IgM antibodies. **(F)** Correlation heatmap between MZ area, density of pIRS1^+^ and pFOXO1^+^ cells in MZ and autoantibody production. Spearman corelation was applied. **(G)** Confocal images of PNA staining of GC and perifollicular. pIRS staining in GC of NT157-treated and control mice. **(H)** Area of PNA^+^ GC. **(I)** Density of pIRS^+^ cells. **(J)** ICOS^+^ CD4^+^ and CXCR5^+^ICOS^+^ cells. **(K)** Box plots of IgG antibodies against RF, CCP, dsDNA. **(L)** Box plots of total IgG and anti-mBSA IgG antibodies in serum of NT157/shIGF1R-treated and control mice. *p<0.05.

To further characterise CD21^+^CD19^+^ B cells that were increased after IGF1R inhibition, we analysed the expression of IgM on CD3^-^ cells and found that IgM was more often present on CD21^+^ cells which defines MZ B cells. In fact, we observed significantly enlarged CD21^+^ and CD23^+^ populations among IgM^+^ B cells (**Figure 3A**). Expression of ICOSL and CXCR5 are important for B cell translocation from the MZ into the follicle and for the acquisition of a germinal centre (GC) phenotype (45). The analysis of CXCR5 on CD21^+^IgM^+^ICOSL^+^ B cells revealed a significant reduction of this population after IGF1R inhibition (**Figure 3B**), which could have negative consequences for the ability of IgM^+^ B cells to migrate from the MZ into the follicles in NT157-treated mice. A similar analysis of CXCR5 was done for the CD23^+^IgM^+^ B cell population. Despite that the CD23^+^IgM^+^ B cell population was enlarged after IGF1R inhibition (**Figure 3A**), we observed no significant change in expression of either ICOSL (**Figure 3B**) or CXCR5 (**Figure 3C**) on these cells. Ergo, the results indicated that IGF1R signalling was of major importance for determining the B cell phenotype, with a transition from MZ to follicular after the treatment. IGF1R inhibition led to an enlargement of MZ by promoting an accumulation of ICOSL^-^IgM^+^CD21^+^B cells.

To assess if IGF1R inhibition affected the B cell function, we measured the level of total IgM and antigen-specific antibodies against mBSA and autoantibodies to double-stranded (ds)DNA, cyclic citrullinated peptides (anti-CCP) and to Fc-region of IgG (known as Rheumatoid Factor, RF) in supernatants of LPS-stimulated splenocyte cultures. We found that IGF1R-deficient spleen cultures had a significant and pleiotropic increase in IgM autoantibody production (**Figure 3D**). The inhibition of IGF1R signalling had a similar stimulating effect on the levels of total IgM, while no difference in the production of antigen-specific anti-mBSA IgM (**Figures 3E**) was observed. We also found that IgM autoantibody levels were positively correlated to the density of pIRS1^+^ and pFOXO1^+^ cells in spleen MZ (**Figures 3F**), which was not found for antibodies to mBSA or total IgM.

### IGF1R inhibition affects T cell function and interaction within the GC

To study if the inhibition of IGF1R signalling affected the formation of intra-follicular germinal centres (GC), we visualised GC area in spleen using peanut agglutinin (PNA) binding to the galactose specific C-type lectins (46). A morphometric analysis of spleen sections revealed that the PNA^+^ area of GC (µm^2^) was increased by 96% in NT157-treated compared to sham-treated mice (p=0.036. **Figures 3G, H**). We found the presence of pS^612^IRS1^+^ cells within the GC area (**Figure 3G**). In fact, the density of pS^612^IRS1^+^ cells within the GC was significantly greater in NT157-treated mice compared to sham-treated controls (**Figure 3I**). Neither pS^612^IRS1^+^ nor pS^256^FoxO1^+^ cells were observed at other locations within the follicles. A thin PNA^+^ belt was clearly visible in the perifollicular area of NT157 treated mice suggesting enrichment for C-type lectin receptor-bearing cells in that area (**Figure 3G**) fortifying the follicular border. These IHC results are supported by flow cytometry which revealed accumulation of a C-type lectin receptor CD23/CLEC4J in CD19^+^ B cells after IGF1R inhibition (**Figure 3A**) and recognised the follicular B cell population.

Since the expression of ICOSL on SSC^hi^APC decreased with IGF1R inhibition (**Figure 2B**), we analysed the expression of its receptor ICOS on CD4^+^T cells. The results showed a 9.6% increase of ICOS^+^CD4^+^ cells with IGF1R intervention (p=0.0040. **Figure 3J**). The observed increase in ICOS^+^CD4^+^ cells largely on CXCR5^-^ cells (p=0.017) and correlated positively with the GC area (r=0.54, p=0.088). We neither observed a change in CXCR5^+^ICOS^+^CD4^+^ cell population (p=0.46) nor in expression of ICOS on CD8^+^ cells (p=0.26). Since insufficient CXCR5 expression on T cells is primarily associated with the follicular Th cell dysfunction in GC resulting in lower class-switching of B cells, we measured IgG antibody production in supernatants from LPS-stimulated splenocyte cultures. Inhibition of IGF1R signal had neither significant effect on the total level of IgG1 nor on the level of anti-mBSA IgG1 in those cultures (**Figure 3L**). However, we did observe significantly lower levels of anti-CCP IgG1 (p=0.041) and somewhat lower anti-dsDNA IgG1 (p=0.097. **Figure 3K**), which reflected a partial delay in antibody class-switching acquired after IGF1R inhibition.

### Low IGF1R expression in CD14^+^ APC was associated with autoantibody production in RA patients

**Figure 4:**
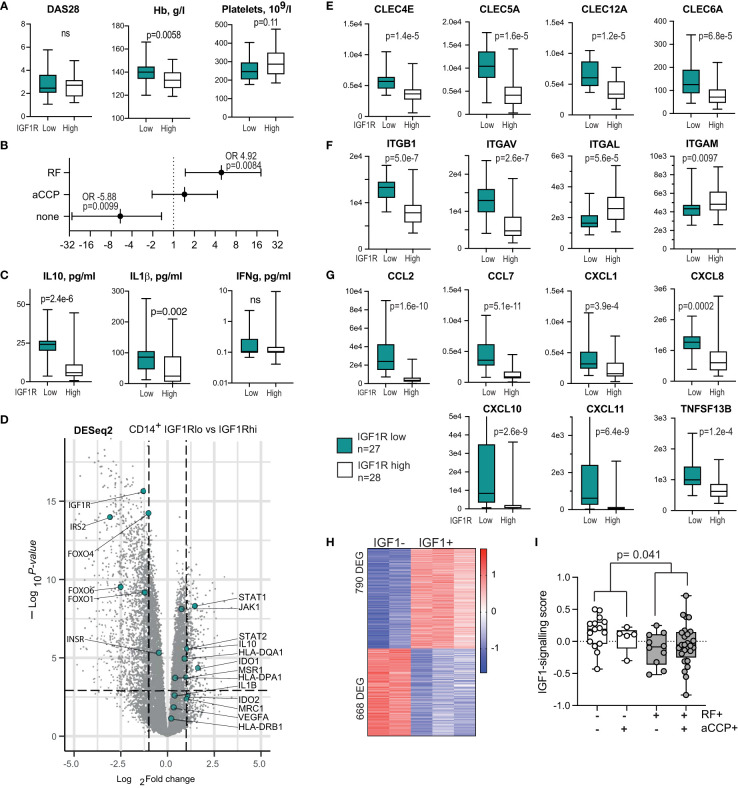
IGF1R-deficient CD14^+^ antigen-presenting cells are prevalent among the autoantibody-producing RA patients. **(A)** Box plots of the disease activity score (DAS28), haemoglobin level and platelet count in RA patients with low (n=27) and high (n=28) IGF1R expression in CD14^+^ cells. **(B)** Forest plot of odds ratio for antibody production between IGF1R^lo^ and IGF1R^hi^ patients. **(C)** Box plots of cytokine levels in supernatants of IGF1R^lo^ and IGF1R^hi^ CD14^+^ cells. **(D)** Volcano plot of whole-genome transcriptomics in IGF1R^lo^ and IGF1R^hi^ CD14^+^ cells, by RNA-seq. **(E)** Box plot of C-type lectin receptors. **(F)** Box plots of integrin expression. **(G)** Box plots of chemokine production. **(H)** Heatmap of the genes differentially expressed in response to IGF-1 stimulation and comprising IGF1-signature score in CD14^+^ cells. **(I)** Box plots of IGF-1-signature score in samples with different pattern of autoantibody production.

To investigate if low IGF1R expression on APC was associated with autoantibody production, we analysed CD14^+^ cells isolated from the blood of 55 RA patients with known autoantibody status (**Table 1**). The choice to use CD14^+^ cells is justified by high prevalence of this group of APC in blood circulation and by high expression density of IGF1R on those cells. Importantly, human circulating CD14^+^ cells are functionally comparable with the MHCII^+^CD11b^+^ spleen macrophages in mice. When the patients were split by the median expression of IGF1R in CD14^+^ cells, it was evident that the IGF1R^lo^ group (n=27) had significantly higher haemoglobin levels and somewhat lower platelet counts compared to the IGF1R^hi^ group, which could indicate a generally non-inflammatory environment of IGF1R^lo^CD14^+^ cells (**Figure 4A**). Notably, the IGF1R^lo^CD14^+^ cells were significantly more frequent in patients producing RA-specific antibodies RF and anti-CCP, while IGF1R^hi^CD14^+^ cells were predominant in antibody-negative RA patients (**Figure 4B**).

To further investigate the consequences of low IGF1R expression in APC, we compared the transcriptional profile of CD14^+^ cells with high and low IGF1R expression, using DESeq2. As expected, IGF1R^lo^CD14^+^ cells had lower expression of several downstream signalling molecules of IGF1R including INSR, IRS2, FOXO4, FOXO1 and FOXO6 proteins (**Figure 4D**), which replicated the suppression of IRS and FOXO signalling found after IGF1R inhibition in mice. Metabolically, IGF1R^lo^CD14^+^ cells relied on the rate limiting catalyser of the essential amino acid tryptophane along the kynurenine pathway IDO1 and IDO2, important for the peripheral immune tolerance and immune responses. This was combined with a significantly higher expression of STAT1, STAT2 and JAK2.

As predicted by the non-inflammatory environment, the IGF1R^lo^CD14^+^ cells produced higher levels of IL10 and IL1β as judged by RNA-seq and by protein cytokine levels in cell culture supernatants (**Figures 4C, D**), a sign of M2-type macrophages. In addition to cytokines, IGF1R^lo^ CD14^+^ cells expressed higher levels of MHCII receptors HLA-DQA1, HLA-DPA1 and HLA-DRB1, and in C-type lectin binding receptors including the macrophage scavenger receptor 1 (MSR1) and the mannose receptor 1 (MRC1) directly interacting with T cells (47) (**Figure 4D**), and CLEC6A/DCIR2, CLEC4E/MINCLE, CLEC5A/MDL1, CLEC12A/MICL, CLEC4A/DCIR (**Figure 4E**) essential for immunoregulation of T and B cell function (48–51).

Adhesion molecules integrins ITGB1 and ITGAV were upregulated, while ITGAL and ITGAM were downregulated on IGF1R^lo^CD14^+^ cells (**Figure 4F**). Additionally, we observed a significant expression difference in C-C family chemokines MCP1/CCL2 and CCL7, and C-X-C chemokines CXCL1, CXCL8, CXCL10 and CXCL11 attracting CXCR3^+^ T and B cells, and BAFF/TNFSF13B required for directing their maturation into plasma cells and GC formation (45, 52) (**Figure 4G**). Taken together, IGF1R^lo^CD14^+^ cells presented the subset of immune regulatory and tolerogenic macrophages (53) that could suppress elimination of autoreactive cells.

To experimentally confirm connection between autoantibody production and insufficient IGF1R signalling, we utilised external RNA-seq dataset of human peripheral blood monocyte-derived macrophages that were stimulated with IGF1 *in vitro* (*GSE202515*). First, we compared transcriptional profile of IGF-1 and vehicle stimulated macrophages and revealed 1458 DEG (790 upregulated and 668 downregulated genes) dependent of IGF1 stimulation (**Figure 4H**). Transcription sum of these DEG comprised the IGF-1 signalling score, which was calculated for each of the CD14^+^ RNA-seq sample of 55 RA patients. Comparison of the IGF-1 signalling score between the samples demonstrated that the score was the lowest in the patients producing both RF and anti-CCP autoantibodies, followed by the RF-positive patients (**Figure 4I**). Thus, using experimentally obtained IGF-1 signalling score, these findings revealed a connection between low IGF1-IGF1R signalling response in CD14^+^ cells and autoantibody production. This was consistent with the direct comparison of the RA patients separated by the median expression of IGF1R. This also reproduced the observations done in mice where IGF1R inhibiting treatment resulted in enrichment with IGF1R^lo^APCs in spleen and a sustained autoantibody production.

## Discussion

In this study, we demonstrate that compromised IGF1R signalling in CD14^+^ cells of RA patients and experimental inhibition of IGF1R signalling in mice has an unexpected effect resulting in break of tolerance and formation of autoreactive B cells producing a pleiotropic set of autoantibodies. This finding distinguishes the anti-inflammatory effect of IGF1R inhibition from its pro-autoimmune consequences. Expression of IGF1R in the peripheral blood leukocytes of RA patients was directly correlated to swollen and painful joint number and high disease activity (16). In experimental arthritis, sufficient IGF1R signalling at the time of antigen challenge enabled the migration of inflammatory cells into the joints. Inhibition of IGF1R signalling abolished T cell migration and alleviated arthritis (16). A similar effect with a reduction of inflammation was achieved by the conditional deletion of IGF1R in CD4^+^ cells in multiple sclerosis (17).

Macrophages is the cell type showing the highest expression of IGF1R and inhibition of IGF1R resulted in the accumulation of MHCII^+^CD11b^+^ APCs in RA patients and in mice. Transcriptional analysis by RNA-seq and cytokine levels in supernatants of CD14^+^ cells allowed to characterise IGF1R^lo^ cells as M2-type. The IGF1R^lo^CD14^+^ cells expressed MHCII, MRC1 and MSR1 receptors important for the immune regulatory arsenal of macrophages (54). Additionally, IGF1R^lo^CD14^+^ cells produced high levels of IL10 and IL1β in supernatants and were in total less inflammatory compared to IGF1R^hi^CD14^+^ cells. High expression of CXC chemokines and the C-type lectin receptors on IGF1R^lo^CD14^+^ cells signal their high affinity towards the C-type lectin expressing PNA^+^ secondary lymphoid structures characteristic for GC reaction, which reproduced the phenotype of APC after inhibition of IGF1R in mice.

In this study, we demonstrate that IGF1R signalling regulates FOXO family of transcription factors, which are central integrators of growth factor signalling, oxidative stress and inflammation (55–57). Inhibition of IGF1R resulted in the extrafollicular enrichment of inactive pFOXO1 in an enlarged MZ area, and both these observations showed an inverse correlation to expression of the FOXO-controlled genes ICOSL and CD62L by APC and B cells. Both, ICOSL and CD62L provide co-stimulatory signal to the T cell receptor acting an important link in communication between APC and the adaptive immunity (56, 58, 59). Similar to our findings, FOXO1 deficient peripheral B cells have been shown to express low levels of CD62L and to fail class-switch recombination (41, 60). Additionally, FOXO1 silencing promoted monocyte expansion, which was concomitant with exacerbation of SLE-like autoimmunity (51). Analogously, IGF1R^lo^CD14^+^ cells from RA patients had low expression of FOXO family proteins, which coincided with a higher frequency of autoantibody production in those patients, which leads us to believe that IGF1R dependent activity of FOXO in APC was an important mechanism counteracting break of immunological tolerance.

This study showed that disrupted signalling through IGF1R contributes to autoreactivity and autoantibody production by playing an important role in B cell trafficking through the MZ into the follicles. Disruption of IGF1R signalling resulted in an accumulation of MHCII^+^APC, an enlarged MZ areas, and a higher splenic frequency of IgM^+^CD21^+^ cells likely retained in the MZ, which predispose to their direct communication. The molecular nature of the direct interaction between MHCII^+^APC and B cells has not been elucidated in depth and requires further studies. The C-type lectins upregulated in IGF1R^lo^CD14^+^ cells of RA patients and in APC after IGF1R inhibition in mice and their emerging immune regulatory function (47, 50, 51) are the candidate mediators of such an interaction.

It has previously been reported that IGF1R is important for an adequate T-cell independent B cell response and that this is connected to their location in the MZ (61, 62). The formation of the MZ B cells is based on the crosslinking BCR by repetitive non-protein epitopes and on the direct interaction with APC. The MZ macrophages have been shown to suppress adaptive immunity. Hence, IGF1R inhibition followed by FOXO inactivation appear to lower the threshold for extrafollicular B cell activation and autoantibody production. This experimental chain of cellular events preceding and permitting development of autoimmunity is prone to expand over the lifetime resembling spontaneous autoimmunity in man (63, 64). Whether T cells were needed as the third part for mediating this communication under the condition of IGF1R inhibition was addressed. It seems likely that the reduced expression of ICOSL on APC and CD19^+^B cells weakened the ability of T cells to interact with and control both B cells and macrophages. Thus, insufficient T cell control in the MZ could have created the conditions for autoimmunity observed after IGF1R inhibition in our study. This hypothesis finds support in the reports that ICOSL deficient mice produced low levels of IgG1 (58) and had the GC reaction disruption at an early stage, which revealed a significant role of ICOSL in Ig class-switching reaction. A similar lowering of Ig class switch was reported in SLE patients treated with anti-ICOSL antibodies (59), but this did not affect IgM production. Interestingly, we observed that development of the autoreactive B cells existed in parallel and did not interfere with conventional T cell dependent antigen response. An antigen challenge with mBSA after IGF1R inhibition led to the ordinary T cell dependent sequence of events including the initial formation of the mBSA-specific B cells producing IgM, formation of GCs, and development of the mBSA-specific class-switched IgG1 producing B cells.

Taken together, the study demonstrates that IGF1R is an important control mechanism that prevent development of autoimmunity. Disruption of IGF1R signalling in human RA and experimental mouse model results in involution of the FOXO pathway, which prolongs communication between macrophages and B cells and, under the condition of insufficient T cell feedback, permits the production of IgM that targets the canonical auto-antigens dsDNA, Fc-portion of IgG, and cyclic citrullinated peptides with well-documented pathogenic potential. IGF1R targeting is the subject of large numbers of clinical interventional studies. In addition to solid tumours and haematological malignancy, antibodies against IGF1R are currently in clinical trials for patients with autoimmune ophthalmopathy related to the Grave’s disease (26, 65), and other thyroid eye diseases (NCT01868997, NCT04583735), diabetic macula oedema (NCT02103283) and diffuse cutaneous systemic sclerosis (NCT04478994). The novel findings presented in this study raise awareness of the break of immunological tolerance and the increased risk of serious clinical consequences following IGF1R inhibition.

## Author contributions

Conceiving the study, MIB, RP, and MB; collecting material, ME, KA, CW, SS, RP, and MIB; Laboratory work, KA, ME, SE, and CW; Statistical analysis, ME, SE, KA, CW, and MIB; drafting the manuscript, MIB, CW, ME, SE, and MB. All authors discussed and helped interpret the data and provided feedback during preparation of the manuscript.

## Funding

This work was supported by the Swedish Research Council [2017-03025 and 2017-00359 to MIB]; the Swedish Association against Rheumatism [R-566961; R-751351 and R-860371 to MIB]; the King Gustaf V:s 80-year Foundation [FAI-2018-0519 and FAI-2020-0653 to MIB]; and the Regional agreement on medical training and clinical research between the Western Götaland county council and the University of Gothenburg [ALFGBG-717681 and ALFGBG-965623 to MIB].

## Conflict of interest

The authors declare that the research was conducted in the absence of any commercial or financial relationships that could be construed as a potential conflict of interest.

## Publisher’s note

All claims expressed in this article are solely those of the authors and do not necessarily represent those of their affiliated organizations, or those of the publisher, the editors and the reviewers. Any product that may be evaluated in this article, or claim that may be made by its manufacturer, is not guaranteed or endorsed by the publisher.
