# Supplementary material for: IGF1R signalling is a guardian of self-tolerance restricting autoantibody production
Source: Front Immunol. 2022 Aug 29;13:958206. doi: 10.3389/fimmu.2022.958206 (PMC9464816; doi:10.3389/fimmu.2022.958206)
Supplement: Supplementary file 1 [file DataSheet_1.doc]

**Table S1. Antibodies used for flow cytometry (and immunohistochemistry)**

| Target | Clone | Source |
| --- | --- | --- |
| CD3-PE | 145-2C11 | BD |
| CD4-PB | RM4–5 | BD |
| CD8-PeCy7 | 53-6.7 | eBiosciences |
| CD11c-PeCy7 | HL3 | BD |
| CD19-APC-H7 | ID3 | BD |
| CD21- APCCy7 | 7E9 | Biolegend |
| CD23- PeCy7 | B3B4 | eBiosciences |
| B220- PerCp | RA3-16B2 | BD |
| F4/80-APCCy7 | BM8 | eBiosciences |
| IgM- V450 | R6-60.2 | BD |
| CXCR5 | SPRCL5 | eBiosciences |
| ICOS-PE | 15F9 | Biolegend |
| ICOSL- PE | 500A2 | BD |
| MHCII-e450 | M5/114.15.2 | Biolegend |
| IGF-1R | D23H3 | Cell Signaling |

**Table X. Antibodies used for immunohistochemistry**

| Target | Isotype | Conjugate | Product number | Source |
| --- | --- | --- | --- | --- |
| pS^612^IRS1 | Rabbit IgG | - | 44-550G | Invitrogen |
| pS^256^FOXO1 | Rabbit IgG | - | PA5-38132 | Invitrogen |
| IgM | Goat F(ab’)_2_ | biotin | 115-067-020 | Jackson Immunoresearch |
| Rabbit IgG | Goat IgG | AF488 | A11034 | Thermo Fisher Scientific |
| - | PNA | biotin | B-1075-5 | Vector laboratories |
| - | streptavidin | AF635 | S32364 | Thermo Fisher Scientific |
| Marco | Rat IgG | - | MCA1849 | BioRad |
| Rat IgG | Donkey IgG | Dylight550 | SA5-10027 | Invitrogen |
| Nuclear stain | Hoechst | - | H21480 | Molecular Probes |

**Table S2. Antibodies used for immunoglobulin ELISA**

| Target | ELISA | Conjugate | Isotype | Product Number and Source |
| --- | --- | --- | --- | --- |
| IgM | mBSA | Biotin | Goat F(ab’)_2_ | 115-066-075, Jackson Immunoresearch |
| IgG | mBSA | Biotin | Goat F(ab’)_2_ | 115-066-071, Jackson Immunoresearch |
| IgG1 | mBSA | Biotin | Goat (γ1 chain spec) | 1070-08, SouthernBiotech |
| IgG2a | mBSA | Biotin | Goat (γ2 chain spec) | 1080-08,  SouthernBiotech |
| IgG2b | mBSA | Biotin | Goat (γ2 chain spec) | 1090-08, SouthernBiotech |
| IgG3 | mBSA | Biotin | Goat (γ3 chain spec) | 1100-08, SouthernBiotech |
| Mouse Igs  ExtrAvidin | Total,  mBSA | Peroxidase |  | E2886, Sigma |

**Table S3. Mouse primers for real time PCR**

| Target | Forward Primer | Exon | Reverse | Exon |
| --- | --- | --- | --- | --- |
| FOXO1 | AGTGGATGGTGAAGAGCGTG | 1 | TGTGAAGGGACAGATTGTGGC | 2 |
| IRS1 | GCCAGAGGATCGTCAATAGC | 1 | AAAGGAGGATTTGCTGAGGTC | 2 |
| IRS2 | ACGAGAGCGAGAAGAAGTGG | 1 | AGGGCGATCAGGTACTTGTG | 1 |
| Marco | GGCACCAAGGGAGACAAAG | 8 | TTTCCAGCATCACCTTTACC | 9 |
